# A thiomolybdate cluster for visible-light-driven hydrogen evolution: comparison of homogeneous and heterogeneous approaches†

**DOI:** 10.1039/d3se01658g

**Published:** 2024-02-01

**Authors:** Samar Batool, Jasmin S. Schubert, Pablo Ayala, Hikaru Saito, Maria J. Sampaio, Eliana S. Da Silva, Cláudia G. Silva, Joaquim L. Faria, Dominik Eder, Alexey Cherevan

**Affiliations:** a TU Wien, Institute of Materials Chemistry Getreidemarkt 9/BC/02 1060 Vienna Austria alexey.cherevan@tuwien.ac.at; b Institute for Materials Chemistry and Engineering, Kyushu University 6-1 Kasugakoen Kasuga Fukuoka 816-8580 Japan; c LSRE-LCM – Laboratory of Separation and Reaction Engineering – Laboratory of Catalysis and Materials, Faculty of Engineering, University of Porto Rua Dr Roberto Frias 4200-465 Porto Portugal; d ALiCE – Associate Laboratory in Chemical Engineering, Faculty of Engineering, University of Porto Rua Dr Roberto Frias 4200-465 Porto Portugal

## Abstract

This study investigates the hydrogen evolution reaction (HER) efficiency of two photosystems incorporating an all-inorganic molecular thiomolybdate [Mo_3_S_13_]^2−^ cluster as a HER catalyst. First, we delve into the performance of a homogeneous [Mo_3_S_13_]^2−^/[Ru(bpy)_3_]^2+^ (Mo_3_/Ru) dyad which demonstrates high turnover frequencies (TOFs) and apparent quantum yields (AQYs) at 445 nm approaching the level of 0.5%, yet its performance is marked by pronounced deactivation. In contrast, a heterogeneous approach involves anchoring [Mo_3_S_13_]^2−^ onto graphitic carbon nitride (GCN) nanosheets through weak electrostatic association with its triazine/heptazine scaffold. [Mo_3_S_13_]^2−^/GCN (Mo_3_/GCN) displays effective H_2_ generation under visible light, with TOF metrics on par with those of its homogeneous analog. Although substantial leaching of [Mo_3_S_13_]^2−^ species from the Mo_3_/GCN surface occurs, the remaining {Mo_3_}-based centers demonstrate impressive stability, leading to enduring HER performance, starkly distinguishing it from the homogeneous Mo_3_/Ru photosystem. Photoluminescence (PL) quenching experiments confirm that the performance of Mo_3_/GCN is not limited by the quality of the inorganic interface, but could be optimized by using higher surface area supports or a higher concentration of [Mo_3_S_13_]^2−^ sites. Our findings showcase complexities underlying the evaluation and comparison of photosystems comprising well-defined catalytic centers and pave the way for developing analogous surface-supported (photo)catalysts with broad use in energy applications.

## Abstract 2

This study investigates the hydrogen evolution reaction (HER) efficiency of two photosystems incorporating an all-inorganic molecular thiomolybdate [Mo_3_S_13_]^2−^ cluster as a HER catalyst.

## Graphical abstract

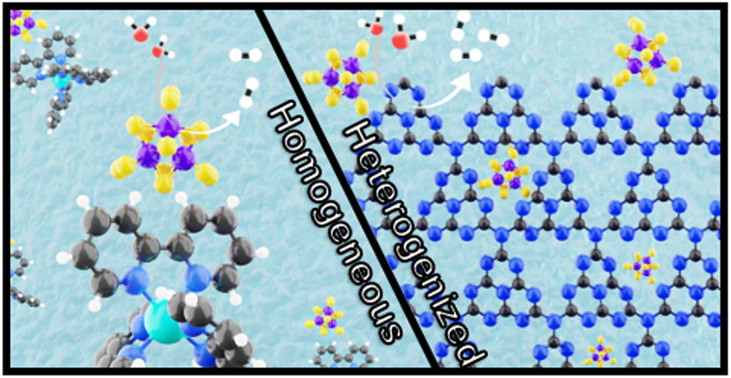

## 1. Introduction

Photocatalysis is often cited as one of the most sustainable and straightforward methods for producing solar fuels from renewable energy resources. Despite decades of research,^1^ however, only a limited number of efficient and stable photosystems for water splitting have been identified,^2,3^ indicating that the path towards developing high-performance photocatalysts is far from straightforward.^4^ As a result, the scientific community continues to search for a set of comprehensive design principles that can guide the synthesis of selective and stable photocatalysts, enabling the efficient conversion of solar energy into chemical energy.^5–8^

Direct capture of sunlight energy and its use to facilitate the desired chemical reaction can be accomplished using either a homogeneous or heterogeneous photosystem that by definition bears pronounced differences in terms of their structure (molecules *vs.* solids), composition (mostly organic *vs.* mostly inorganic), solubility characteristics (molecular solutions *vs.* suspensions), underlying charge transfer and separation processes as well as the nature of catalytic sites. Many homogeneous photocatalytic systems show high turnover frequencies (TOFs); however – compared to their heterogeneous counterparts – often suffer from low turnover numbers (TONs) on account of photosensitizer degradation, catalyst self-aggregation, or formation of colloidal oxide species. Heterogeneous photocatalysts, on the other hand, benefit from adjustable absorption characteristics and superior stability under harsh redox conditions of the photocatalytic reaction but face complications associated with their poorly defined catalytic surface, which limit the degree of control over the processes of charge extraction and interfacial charge transfer.

In light of these challenges and prospects, a hybrid approach that combines the advantages of both homogeneous and heterogeneous photocatalytic systems emerges as a promising solution. Several important avenues for this combination have been explored by the community over the past few decades. One example involves single-metal-site and single-metal-atom catalysts which allow the creation of robust all-inorganic photosystems with unparalleled atom-utilization efficiency.^9–11^ Another strategy exploits stabilization of well-defined molecular catalysts on the surface of solid-state (photoactive) supports, such as in the case of the Co-based (Co_4_O_4_) water oxidation catalyst (WOC) recently embedded within the pores of a rigid coordination network,^12^ or in the field of surface organometallic chemistry which relies on the immobilization of complexes onto structurally controlled inorganic surfaces.^13,14^ Additionally, metal–organic frameworks (MOFs) can be regarded as a promising hybrid photosystem due to their ability to merge molecular units (ligands and metal nodes) into a crystalline solid-state material, thus incorporating the benefits of both molecular and inorganic approaches to (photo)catalysis.^15^ Despite these and many other combinations of molecular and solid-state photosystems that have been explored, only rare studies were able to directly compare the photocatalytic performance of similar photocatalytic systems under homogeneous and heterogeneous conditions.^16^ Nevertheless, by investigating the effects of molecular catalyst immobilization, one can gain insights into the factors that contribute to or restrict the performance of heterogenized catalysts. This type of investigation enables a more comprehensive understanding of the underlying mechanisms and provides valuable information for the design and optimization of highly efficient and stable hybrid photosystems.

Aiming to complement this knowledge gap, we turned our attention to an inorganic thiometalate cluster [Mo_3_S_13_]^2−^, which recently emerged as a promising noble-metal-free catalyst for the hydrogen evolution reaction (HER).^17^ This trinuclear cluster features all the advantages of a molecular catalyst – including the defined geometry, structure and composition – which together allow the shedding of light on its active sites and elucidate the reaction mechanisms.^18–20^ Over the past few years, examples of [Mo_3_S_13_]^2−^ immobilization onto visible-light-active carbon nitride have been reported confirming the ability of surface-attached [Mo_3_S_13_]^2−^ to act as a HER co-catalyst.^21,22^ Additionally, two recent studies reported that the encapsulation of [Mo_3_S_13_]^2−^ clusters into porous heterogeneous scaffolds – such as covalent organic frameworks (COFs)^23^ and MOFs^24^ – yield stable photocatalytic systems, which further emphasizes the benefits of the heterogenization strategy. Most recently, our group reported surface-anchoring of [Mo_3_S_13_]^2−^ on a carbon-free support, *i.e.* TiO_2_, which involved covalent binding and resulted in high and stable HER rates comparable to those of benchmark Pt/TiO_2_.^25^

This work sets out to provide a more comprehensive understanding of the benefits and limitations of heterogenization strategy. Specifically, we aim to directly compare the activity, stability, and other important performance indicators of surface-anchored [Mo_3_S_13_]^2−^ clusters with their performance towards the HER under strictly homogeneous conditions. Our data reveal that despite [Mo_3_S_13_]^2−^ being able to deliver excellent HER performance under homogeneous conditions, strong and rapid deactivation is imminent for this photosystem. In contrast to this, heterogenized [Mo_3_S_13_]^2−^ shows much more stable HER performance with no apparent cluster degradation. Our photoluminescence spectroscopy quenching studies further compare redox pathways in both the photosystems and quantitatively compare their charge transfer kinetics. The data reveal that the performance of the heterogenized [Mo_3_S_13_]^2−^ can be strongly limited by the degree and effectiveness of the electron–hole separation and that an increase in the [Mo_3_S_13_]^2−^ loading or support surface area available for cluster immobilization may play a crucial role in unravelling full potential of this and other relevant thiomolybdate catalysts.^20^

## 2. Results and discussion

**Fig. 1:**
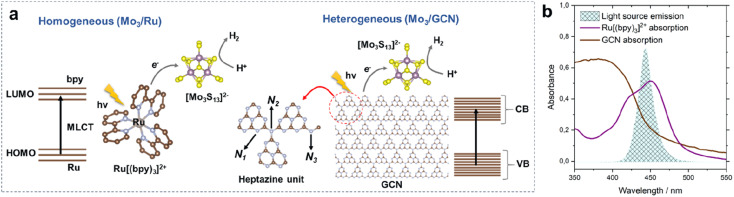
Photocatalytic HER mechanisms on (a) Mo_3_/Ru (homogeneous) and Mo_3_/GCN (heterogeneous) photosystems (brown: C; blue: N; purple: Mo; yellow: S) showing their main components as well as the photoexcitation and charge transfer pathways; LUMO and HOMO stand for the lowest unoccupied and the highest occupied molecular orbitals of [Ru(bpy)_3_]^2+^; CB and VB stand for the conduction and valence bands of GCN; N_1_, N_2_ and N_3_ correspond to the three types of N atoms building the heptazine unit of the GCN framework and (b) absorption spectra of [Ru(bpy)_3_]^2+^ (in methanol, 10^−5^ M) and GCN (*via* diffuse-reflectance spectroscopy, DRS, details in the Experimental section) used to construct the photosystems overlaid with the emission spectrum of a narrow-band LED lamp (center wavelength of 445 nm, details in the Experimental section) used in the photocatalytic studies.

We start by describing two photosystems – a homogeneous and a heterogeneous – involving a thiomolybdate [Mo_3_S_13_]^2−^ cluster as an HER catalyst. On one hand, we evaluate HER performance of the cluster under strictly homogeneous conditions – denoted here as Mo_3_/Ru – which involves the use of a state-of-the-art molecular dye, tris(bipyridine)ruthenium(ii) ([Ru(bpy)_3_]^2+^), as a visible-light photosensitizer (Fig. 1a left).^18,26^ On the other hand, we explore the performance of the heterogenized [Mo_3_S_13_]^2−^ cluster following its deposition on thermally exfoliated graphitic carbon nitride (GCN, details on synthesis are in the Experimental section). This photosystem is denoted here as Mo_3_/GCN. As shown in Fig. 1a (right), GCN sheets play the role of the light absorber (*i.e.* photosensitizer), while the [Mo_3_S_13_]^2−^ clusters anchored on the surface act as a formal co-catalyst,^21,22^*i.e.* they extract the photoexcited charge carriers from the GCN bulk and promote the reaction of interest by reducing H^+^ to H_2_. Compared to our previous work in which [Mo_3_S_13_]^2−^ was anchored onto a titania surface,^25^ the use of narrow-band gap GCN enables absorption of visible-light photons and thus allows a direct comparison of Mo_3_/GCN with the homogeneous visible-light-driven Mo_3_/Ru photosystem (Fig. 1b and Section 2 ESI†).

The heterogeneous Mo_3_/GCN will be first described from the point of view of its composition and structure. Following this, the photocatalytic HER results – in terms of activity, mechanism and stability – of Mo_3_/Ru and Mo_3_/GCN will be presented and compared aiming to reveal factors that control or limit their catalytic performance.

### 2.1 Heterogenized Mo_3_/GCN photosystem

Na_2_[Mo_3_S_13_] and graphitic carbon nitride (GCN) were synthesized according to previously established methods (see the Experimental section) and their purity and structure were confirmed using powder X-ray diffraction (XRD) and IR spectroscopy (Fig. S1†). Diffuse reflectance spectroscopy (DRS) of the as-obtained GCN nanosheets reveals pronounced absorption in the visible range (absorption tail beyond 500 nm) corresponding to an expected optical band gap of 2.75 eV (Fig. S2a and b†), which confirms the capability of the supporting GCN to absorb in the range of the molecular [Ru(bpy)_3_]^2+^ sensitizer.^27^ The [Mo_3_S_13_]^2−^/GCN composite (Mo_3_/GCN) was synthesized following a wet-impregnation route (for more details see the Experimental section). Compared to the DRS spectrum of bare GCN, the DRS spectra of Mo_3_/GCN show an additional broad band centered at 456 nm (Fig. S3†), which can be ascribed to the characteristic ligand-to-metal charge transfer transition of [Mo_3_S_13_]^2−^ suggesting successful deposition of [Mo_3_S_13_]^2−^ onto the GCN surface.^28^ Total reflection X-ray fluorescence spectroscopy (TXRF) further confirms the presence of Mo in the composite and allows the estimation of the real [Mo_3_S_13_]^2−^ loading to be around 3.9 wt% (Table 1, details in the Experimental section). Interestingly, this value is significantly lower compared to the intended loading of 10 wt% and – in contrast to oxide-based supports^25^ – indicates a weaker nature of the interaction between organic GCN and [Mo_3_S_13_]^2−^. A similar actual-to-expected loading is also attained for a 1 wt% nominal loading value (Table 1), which further corroborates that the adsorption of [Mo_3_S_13_]^2−^ on GCN is not governed by the presence of suitable adsorption sites on the GCN surface, but is rather defined by the adsorption/desorption equilibrium.

**Table 1:** Quantification of real loadings of the [Mo_3_S_13_]^2−^ cluster in Mo_3_/GCN composites derived from TXRF data

Composites	Nominal loadings [Mo_3_S_13_]^2−^ (wt%)	Real loadings [Mo_3_S_13_]^2−^ (wt%)
Mo_3_/GCN	10	3.9
	1	0.36
Mo_3_/H-GCN	10	5.1

**Fig. 2:**
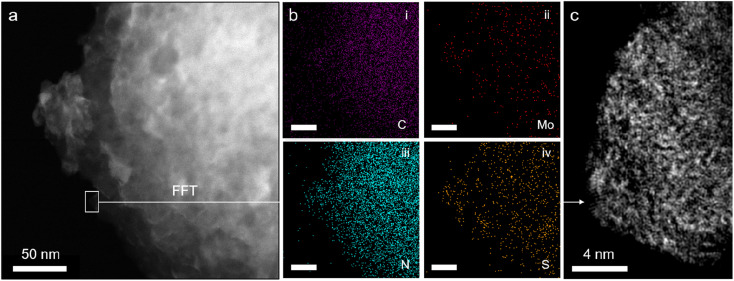
STEM micrographs of the Mo_3_/GCN composite (a) high-resolution high-angle annular dark field (HAADF) STEM image of the Mo_3_/GCN composite showing an aggregate of GCN nanoflakes along with its (b) EDS-derived elemental mappings featuring the distribution of (i) C, (ii) Mo, (iii) N, and (iv) S elements, and (c) FFT-transformed magnified region of one of the GCN flake's edges (see selected area in (a)) showing collections of bright spots that correspond to the heavy Mo atoms.

Attenuated total reflectance Fourier transform infrared spectroscopy (ATR-FTIR) spectra of pristine GCN (Fig. S4†) match well with those in the literature, confirming the formation of a heptazine-based scaffold (Fig. 1a, right).^27^ Notably, the results for Mo_3_/GCN composites show no vibrations corresponding to [Mo_3_S_13_]^2−^, as expected from the low cluster loadings. Therefore, scanning transmission electron microscopy (STEM) was used to provide evidence for the presence of [Mo_3_S_13_]^2−^ on the GCN surface. Energy dispersive X-ray (EDX) maps acquired on the nanoscale (Fig. 2a and b) confirm chemical identity of the elements and reveal an even distribution of Mo and S over the entire surface of the GCN aggregates. High-resolution fast-Fourier transformed (FFT) image in Fig. 2c focuses on the edge of a typical GCN flake and shows the presence of multiple bright spots. Considering the high *Z*-contrast between Mo and C/N atoms, these spots likely correspond to heavy Mo atoms dispersed on GCN and their high density further indicates close proximity of the clusters. Interestingly, a closer look at Fig. 2c also reveals the formation of chain-like structures suggesting the possibility of partial cluster oligomerization, in line with MoS_*x*_-based nanostructuring observed on similar carbon-based surfaces.^29^

**Fig. 3:**
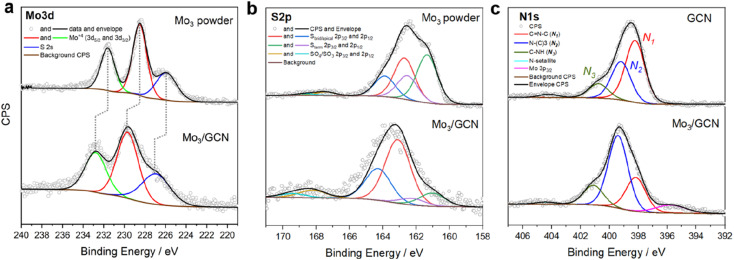
XPS spectra of the Mo_3_/GCN cluster. The [Mo_3_S_13_]^2−^ clusters before (Mo_3_ powder) and after (Mo_3_/GCN) attachment to the GCN surface, (a) Mo 3d, (b) S 2p, and (c) N 1s (N_1_: pyridinic nitrogen; N_2_: quaternary nitrogen; N_3_: secondary amine), with corresponding fits.

Surface-sensitive X-ray photoelectron spectroscopy (XPS) was further used to verify the structure of [Mo_3_S_13_]^2−^ after attachment. The survey spectra confirm the presence of all elements expected from the Mo_3_/GCN composition (Fig. S5†). The detailed Mo 3d profile of Mo_3_/GCN in Fig. 3a only shows peaks corresponding to Mo^4+^ with an overall profile that resembles that of the free [Mo_3_S_13_]^2−^ cluster. This indicates the preference for a trinuclear structure of the cluster cores and further suggests that neither oxidation nor decomposition of the central {Mo_3_} units of the cluster occurred upon anchoring. Importantly, we also observe a shift of the Mo 3d peaks maxima by 1.2 eV to higher binding energies, which is indicative of electronic interactions involving charge transfer from the cluster to the support, in line with the anionic nature of [Mo_3_S_13_]^2−^. The S 2p signal profile of Mo_3_/GCN undergoes a more significant change upon attachment, which suggests a restructuring of the S-containing ligands. A closer look at the deconvoluted profiles (Fig. 3b) indicates partial loss of bridging disulfides, which can be linked to the partial transformation of the clusters into more complex MoS_*x*_ fragments, in line with cluster oligomerization observed in STEM.^30^ Detailed analysis of the N 1s edge (Fig. 3c) further allows evaluation of the nature of the cluster binding and the degree of [Mo_3_S_13_]^2−^/GCN interactions from the support point of view. In the case of the Mo_3_/GCN composite, we observe a strong drop in the major C=N–C component (N_1_) and a concurrent increase of the quaternary N–(C)_3_ signal (N_2_), which suggests that the cluster attachment causes the disruption of the GCN framework – specifically its heptazine/triazine units – in line with the electrostatic binding model (Fig. 1a, right). Overall, the data suggest successful heterogenization of high-density thiomolybdates on the surface of exfoliated GCN nanosheets and confirm their homogeneous deposition along with the intact nature of their {Mo_3_} cores.

### 2.2 Optimization of HER conditions

#### 2.2.1 Homogeneous photosystem

We first established experimental HER protocols for the Mo_3_/Ru molecular catalyst/absorber dyad. Two studies have recently reported photocatalytic HER performance of NH_4_[Mo_3_S_13_] clusters under homogeneous conditions, which laid the foundation for our study. Dave *et al.*^18^ performed visible-light-driven HER experiments using a custom-built reactor filled with 2 mL of the reaction mixture consisting of 0.3 μM [Mo_3_S_13_]^2−^ as a catalyst, 20 μM [Ru(bpy)_3_]^2+^ as a sensitizer and 0.1 M ascorbic acid (H_2_A) as an electron donor using methanol : water (MeOH : H_2_O, 9 : 1) as a mixed solvent system. In parallel, Lei *et al.*^26^ evaluated HER performance of [Mo_3_S_13_]^2−^ with [Ru(bpy)_3_]PF_6_ as a sensitizer, using 0.1 M of H_2_A as a sacrificial agent in an acetonitrile : water (ACN : H_2_O, 9 : 1) solvent mixture. Considering the relatively similar set of conditions reported in these studies, we first validated our Mo_3_/Ru photosystem by using both MeOH : H_2_O and CH_3_CN : H_2_O solvent mixtures with various sensitizer-to-catalyst concentration ratios: 65-to-1 (similar to conditions by Dave *et al.*^18^) and 13-to-1 (similar to conditions by Lei *et al.*) – details are in the Experimental section. Table S1† shows that the Mo_3_/Ru couple could generate H_2_ in both solvent systems with apparent quantum yield (AQY) values of up to 0.5%, whereas a 5-fold decrease in the [Mo_3_S_13_]^2−^ concentration only resulted in a 2-fold drop in the H_2_ amount generated. This non-correlated behavior indicates that the HER performance of this homogeneous photosystem is rather limited by light capture efficiency *i.e.* photosensitization.

Next, aiming for the ultimate comparison of homogeneous Mo_3_/Ru and heterogenized Mo_3_/GCN samples, apart from ascorbic acid previously employed as an electron donor, we also considered methanol (MeOH, being a representative alcohol) and triethanolamine (TEOA, being a representative tertiary amine) commonly used as sacrificial agents in the literature (details in Section 3 in the ESI†). Two important results can be highlighted. Firstly, since the use of the MeOH/H_2_O solvent mixture originally proposed by Dave *et al.* yielded much higher HER performance of the Mo_3_/Ru couple (4-fold increase), we aimed to verify if MeOH additionally acts as an electron and a proton donor.^31^ HER experiments in MeOH solutions with and without the addition of a sacrificial donor (H_2_A) in Fig. S6† show that no H_2_ could be generated in the absence of H_2_A, confirming that MeOH acts only as a solvent. Secondly, we observed that the replacement of H_2_A with TEOA yielded no H_2_ (see Table 2), which we relate to the inability of [Ru(bpy)_3_]^2+^ to oxidize the amine due to its low-lying redox potential. Based on these results, we continue the benchmarking of the Mo_3_/Ru performance obtained from methanolic H_2_A solutions.

**Table 2:** Amount of H_2_ produced by [Mo_3_S_13_]^2−^ in homogeneous (Mo_3_/Ru) and heterogeneous (Mo_3_/GCN) photosystems after 60 min of visible light illumination with 445 nm. Turnover frequencies (TOFs) are calculated based on the real [Mo_3_S_13_]^2−^ loadings derived from TXRF (Table 1). Blank experiments in the absence of [Mo_3_S_13_]^2−^ resulted in a negligible (<0.1 nmol) amount of H_2_ generated

Phase	Catalyst	Sacrificial donor	Solvent	Photosensitizer	H_2_ (nmol)	TOF (min^−1^)
Homogeneous	[Mo_3_S_13_]^2−^50 μM	H_2_A 0.1 M	MeOH : H_2_O (9 : 1)	[Ru(bpy)_3_]PF_6_	390	0.065
		TEOA 0.1 M		0.645 mM	<0.1	—
Heterogeneous	Mo_3_/GCN 10 wt%	H_2_A 0.1 M	H_2_O	None	52	0.062
		TEOA 0.1 M			197	0.237
	Mo_3_/GCN 1 wt%	TEOA 0.1 M			9	0.117
	Mo_3_/H-GCN 10 wt%	TEOA 0.1 M			241	0.221

#### 2.2.2 Heterogenized photosystem

The HER performance of Mo_3_/GCN was evaluated with an identical photocatalytic setup (description in the Experimental section) using 0.5 mg mL^−1^ of Mo_3_/GCN photocatalyst suspensions in water. The choice of the sacrificial agent, however, becomes a crucial aspect for the comparison: while our data show that H_2_A yields the highest HER rates for Mo_3_/Ru under homogeneous conditions (Fig. S6†), the overwhelming majority of literature employs tertiary amines (such as triethanolamine, TEOA) for GCN-based photocatalysts to allow for efficient hole scavenging at the GCN/solution interfaces. On one hand, TEOA acts as an amphiphilic surfactant allowing bridging the relatively non-polar surface of GCN with water molecules;^32^ on the other hand, it helps the dispersion of GCN nanostructures (often two-dimensional sheets) in the solution,^33^ which in turn facilitates the extent of charge separation and transfer. In view of these factors, for further benchmarking, the HER performance of Mo_3_/GCN is discussed using both sacrificial agents: H_2_A and TEOA.

### 2.3 Comparison of both photosystems

#### 2.3.1 HER performance

Table 2 provides an overview of the photocatalytic performance of homogeneous Mo_3_/Ru and heterogeneous Mo_3_/GCN. While substantially more H_2_ could be generated by [Mo_3_S_13_]^2−^ under homogeneous conditions by using H_2_A (390 nmol h^−1^*vs.* 52 nmol h^−1^, for Mo_3_/Ru and Mo_3_/GCN, respectively), both conditions reveal similar activity per thiomolybdate cluster when considering the number of [Mo_3_S_13_]^2−^ species present (TOF_Mo_3_/Ru_ is 0.065 min^−1^ and TOF_Mo_3_/GCN_ is 0.062 min^−1^). This result shows that heterogenized [Mo_3_S_13_]^2−^ clusters are able to deliver HER performance on par with that of their homogeneous counterpart. The use of TEOA for the Mo_3_/GCN composite – as a more reactive electron donor – further results in a substantial boost in its TOF values reaching as much as 0.237 min^−1^, which suggests that the HER performance of the Mo_3_/GCN composite could be limited by the inefficient (slow) scavenging of the holes photoexcited in GCN rather than by H^+^ reduction.

#### 2.3.2 Limiting factors

We next probed other factors that could be in control of the HER performance of the Mo_3_/GCN composite by examining the impact of [Mo_3_S_13_]^2−^ loading and its attachment strength on the photocatalytic activity. First, we compared the HER performance of Mo_3_/GCN samples loaded with 3.9 and 0.36 wt% of [Mo_3_S_13_]^2−^ (refer to Table 1 for nominal values) and revealed that the higher loading yielded a 22-fold increase in H_2_ amounts generated (*i.e.* 197 *vs.* 9 nmol). This strong activity-loading correlation suggests that the overall performance of the heterogeneous Mo_3_/GCN is restricted by the number of catalytic sites – [Mo_3_S_13_]^2−^ – available on the GCN surface. Second, we prepared a composite using protonated GCN (H-GCN) with a positively charged surface which offers more favorable interactions with the anionic [Mo_3_S_13_]^2−^ (for more details, see Methods).^21,34,35^ As expected, impregnation of H-GCN with [Mo_3_S_13_]^2−^ resulted in 5.1 wt% thiomolybdate loading, a 30% increase compared to the case of bare GCN (3.9 wt%, see Table 1). Nevertheless, when normalizing HER performances of both composites to the number of [Mo_3_S_13_]^2−^ present (Table 2), similar TOF values are obtained for Mo_3_/GCN (0.237 min^−1^) and Mo_3_/H-GCN (0.221 min^−1^). This strongly suggests that the kinetics of the interfacial charge transfer from GCN to [Mo_3_S_13_]^2−^ is not a factor that restricts charge separation and utilization.

These performance-related findings indicate that: (a) the number of electrons taking part in H^+^ reduction (*i.e.* the HER) is directly proportional to the amount of [Mo_3_S_13_]^2−^ on the surface, suggesting that the overall HER performance can be improved by having more [Mo_3_S_13_]^2−^ sites present. (b) The transfer of photoexcited electrons from GCN to [Mo_3_S_13_]^2−^ is rapid with respect to H^+^ reduction (*i.e.* the HER) and is not improved when a stronger [Mo_3_S_13_]^2−^ to GCN interaction/interface is created. (c) The effective scavenging of photoexcited holes can be seen as a performance-limiting factor, which needs to be addressed possibly by introducing other sacrificial agents or appropriate oxidation co-catalysts to aid in efficient charge extraction, separation and utilization.

#### 2.3.3 HER mechanism

**Fig. 4:**
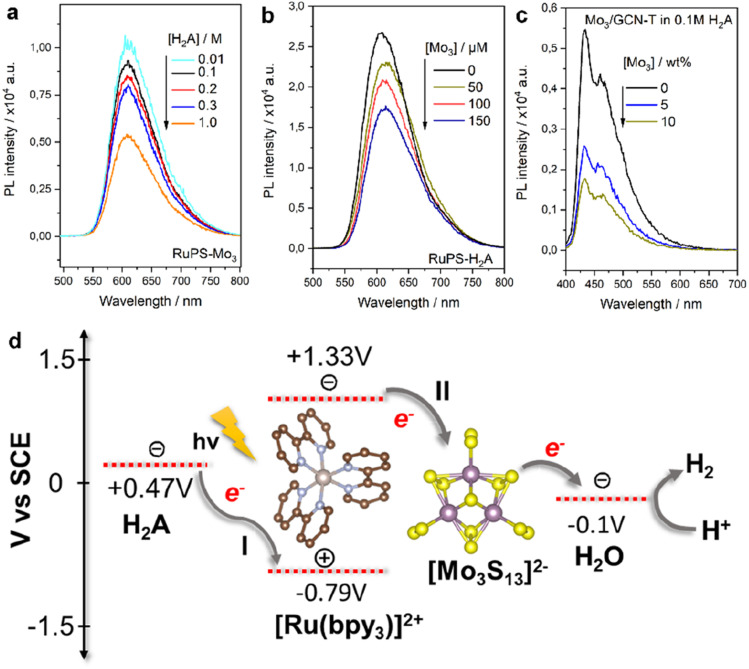
PL emission spectra of the Mo_3_/Ru system with varying (a) H_2_A and (b) [Mo_3_S_13_]^2−^ concentrations and (c) Mo_3_/GCN system with varying [Mo_3_S_13_]^2−^ loadings and (d) schematic of reductive (I) and oxidative (II) quenching mechanisms of the [Ru(bpy)_3_]^2+^ photosensitizer in the presence of the [Mo_3_S_13_]^2−^ catalyst and H_2_A sacrificial donor. Energy levels are based on the relevant literature source.^26^

Photoluminescence (PL) emission spectroscopy quenching studies were conducted to get further insights into the mechanism of the photocatalytic HER using Mo_3_/Ru and Mo_3_/GCN systems. For the Mo_3_/Ru photosystem, the addition of different amounts of H_2_A quenched the PL emission of [Ru(bpy)_3_]^2+^ (Fig. 4a) with a rate constant (*K*_q,red_) of 1.2 × 10^7^ M^−1^ s^−1^ when calculated from a linear Stern–Volmer fitting assuming the dynamic quenching mechanism (Fig. S8a†). This is in line with H_2_A acting as an electron donor as its ascorbate anion (HA^−^) can reductively quench the excited *[Ru(bpy)_3_]^2+^ to [Ru(bpy)_3_]^+^. When using different concentrations of [Mo_3_S_13_]^2−^ (Fig. 4b), we can also observe oxidative quenching of the excited state of *[Ru(bpy)_3_]^2+^ with a rate constant (*K*_q,ox_) of 1.8 × 10^10^ M^−1^ s^−1^ (Fig. S8b†), which is three orders of magnitude higher than that measured for H_2_A. Since our photocatalytic system includes both H_2_A (1 mM) and [Mo_3_S_13_]^2−^ (50 μM), both oxidative and reductive quenching mechanisms (I and II in Fig. 4d) take part in the electron transfer processes. However, given the much higher *K*_q,ox_ compared to *K*_q,red_, the oxidative quenching (extraction of the electron by the thiomolybdate cluster) is likely to dominate the process under turnover conditions, which can be in part explained by the strong electrostatic interaction (ion pairing) between cationic [Ru(bpy)_3_]^2+^ and anionic [Mo_3_S_13_]^2−^. These data allow the following mechanism for the Mo_3_/Ru photosystem (Fig. 4d) to be suggested: the excited *[Ru(bpy)_3_]^2+^ state formed after light absorption undergoes rapid oxidative quenching by the transfer of electrons to the [Mo_3_S_13_]^2−^ catalyst where H^+^ are reduced to H_2_. Meanwhile, the oxidized [Ru(bpy)_3_]^3+^ is reductively quenched to its ground state by the H_2_A present in solution, thereby completing the catalytic cycle.

For Mo_3_/GCN, PL spectra of the composites feature two absorption maxima at around 440 and 480 nm that correspond to the π–π* and n–π* transitions within GCN (Fig. 4c).^27^ Compared to the emission of bare GCN, the deposition of [Mo_3_S_13_]^2−^ leads to a strong PL quenching which can be associated with the facilitated charge separation that highlights the role of [Mo_3_S_13_]^2−^ as a reductive co-catalyst capable of effective extraction of photoexcited electrons. Following the idea of concentration-dependent quenching studies of our Mo_3_/Ru, we also observed that heterogenized Mo_3_/GCN composites with different loadings of [Mo_3_S_13_]^2−^ exhibit different emission intensities. As can be seen in Fig. 4c, increasing the cluster content from 5 wt% to 10 wt% (nominal values) results in more pronounced PL quenching indicating that charge transfer at the [Mo_3_S_13_]^2−^/GCN interface is further facilitated when a higher number of [Mo_3_S_13_]^2−^ is present at the surface. Analyzing the Mo_3_/GCN quenching datasets based on three [Mo_3_S_13_]^2−^ loading values – and assuming that the Stern–Volmer relationship can be applied to describe the kinetics of the GCN quenching – yields a quenching constant *K*_q,ox_ of around 4.1 × 10^7^ M^−1^ s^−1^ (Fig. S8c†). This value is several orders of magnitude lower than the one obtained for the homogeneous Mo_3_/Ru case (1.8 × 10^10^ M^−1^ s^−1^), which is expected for the solid/liquid Mo_3_/GCN interface. In conjunction with the HER discussion above, these insights suggest that the use of photoactive supports with higher surface areas available for [Mo_3_S_13_]^2−^ immobilization could lead to further optimization of the Mo_3_/GCN photosystem's performance.

#### 2.3.4 Stability under turnover conditions

**Fig. 5:**
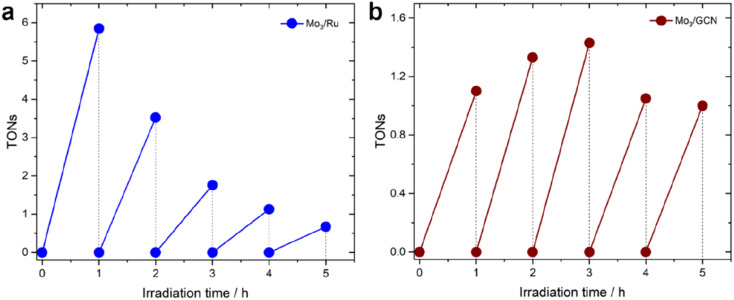
Long-term HER profiles (in H_2_A) of (a) Mo_3_/Ru system and (b) Mo_3_/GCN system. TONs for the Mo_3_/GCN system were calculated based on real loadings of [Mo_3_S_13_]^2−^ clusters on GCN mentioned in Table 1. The experiments were conducted using an LED 445 nm lamp (see Methods section 4.3 for more details).

Long-term visible-light-driven HER experiments were performed using Mo_3_/Ru and Mo_3_/GCN photosystems (details in the Experimental section). Fig. 5a reveals that the homogeneous Mo_3_/Ru couple, despite exhibiting outstanding HER performance (*i.e.* high TOF values) at the initial stage of the illumination, experiences strong and rapid deactivation, which leads to an 8-fold drop in activity after 5 hours. Detailed re-loading experiments confirm that this deactivation is not related solely to the photosensitizer or sacrificial agent depletion, but rather indicates gradual degradation of the [Mo_3_S_13_]^2−^ species (for more details, see ESI Section 5 and Fig. S9†).

In contrast to this, the heterogenized Mo_3_/GCN (Fig. 5b) shows no pronounced deactivation, but rather stable performance – despite with lower average TOFs – over the time course of 5 hours of illumination. XPS of the Mo_3_/GCN composites recovered after the HER run indicate that partial loss (dissolution/leaching) of the clusters takes place during the reaction, which is also suggested by the recovered C=N–C contribution of GCN observed in the N 1s edge (see Fig. S10† for more details). This result is further in agreement with elemental analyses using TXRF (Table S2†), which indicates that only 50% of the original [Mo_3_S_13_]^2−^ species are present in Mo_3_/GCN after the HER. In view of the relatively stable HER performance observed for the Mo_3_/GCN composites (Fig. 5b), however, we can propose that this partial leaching of the [Mo_3_S_13_]^2−^ takes place at the initial stages of the reaction and does not affect long-term HER performance. In fact, detailed Mo 3d and S 2p XPS profiles look qualitatively similar to those of the as-prepared Mo_3_/GCN composite (Fig. S10†), suggesting that no transformation of the surface-bound Mo_3_-species takes place. These remaining [Mo_3_S_13_]^2−^ clusters (1.7 wt% compared to the original 3.9 wt% loading) likely constitute stable and active catalytic sites for the HER that are able to effectively extract the electrons photogenerated in the GCN support and drive stable H_2_ generation.

## 3. Conclusions

Here, we compared HER performances of homogeneous and heterogeneous photosystems involving an all-inorganic molecular [Mo_3_S_13_]^2−^ cluster as an HER site. On one hand, we explored a prototypical homogeneous Mo_3_/Ru couple in which visible light excitation leads to a rapid oxidative quenching of *[Ru(bpy)_3_]^2+^ (PS*) by the anionic [Mo_3_S_13_]^2−^. When combined with a H_2_A hole scavenger, this photosystem delivered high TOFs and AQY_445_ values up to 0.5%; it however suffered from rapid photo-degradation. On the other hand, we demonstrated that [Mo_3_S_13_]^2−^ can be immobilized onto the surface of GCN by relying on electrostatic interactions with its triazine/heptazine framework. We show that this heterogenized Mo_3_/GCN photosystem could generate H_2_ under visible light with TOF values close to those measured for its homogeneous counterpart. Despite significant leaching of the [Mo_3_S_13_]^2−^ species from the surface of Mo_3_/GCN, the rest of the {Mo_3_}-based centers remain structurally intact. They are able to deliver stable long-term HER performance, which is in strong contrast to that of Mo_3_/Ru, which showed photodegradation after 1 hour of photocatalytic reaction. Mechanistic and photoluminescence quenching studies further demonstrated that the performance of Mo_3_/GCN may still be largely limited by the extent of the Mo_3_/GCN interface and the efficiency of electron–hole separation. These results provide valuable insights into the challenges in activity evaluation and comparison for photosystems that involve well-defined catalytic centers (be it clusters or single-metal-atoms or sites), which will help develop similar surface-supported (photo)catalysts for various energy applications.

## 4. Experimental section

### 4.1 Chemicals

The chemicals used for the synthesis were obtained from commercial suppliers and include ammonium molybdate tetrahydrate ((NH_4_)_6_[Mo_7_O_24_]·4H_2_O, Sigma-Aldrich, 99.98% trace metals basis), hydroxylamine hydrochloride (NH_2_OH·HCl, Sigma-Aldrich, 98.0%, ACS Reagent), ammonium sulfide solution ((NH_4_)_2_S_*x*_, Sigma-Aldrich, 20 wt% in H_2_O), carbon disulfide (CS_2_, Sigma-Aldrich, 99.9%), sodium chloride (NaCl, Carl Roth), sodium hydroxide (NaOH, Carl Roth) and dicyandiamide (C_2_H_4_N_4_, 99%, Sigma-Aldrich). The solvents used for the synthesis were deionized water, ethanol (EtOH, from Chem-Lab NV), HPLC-gradient grade methanol (MeOH, from VWR), diethyl ether (from Sigma-Aldrich), and *N*,*N*-dimethylformamide (DMF, from Acros Organics, 99.8%, extra dry over the molecular sieve, acroseal).

### 4.2 Synthetic procedures

#### 4.2.1 Synthesis of (NH_4_)_2_[Mo_3_S_13_]

Following the original studies,^36^ we developed microwave-assisted synthesis where 250 mg of ammonium molybdate tetrahydrate ((NH_4_)_6_[Mo_7_O_24_]·4H_2_O) and 187.5 mg hydroxylamine hydrochloride (NH_2_OH·HCl) were added to 5 mL of ammonium sulfide solution in a 30 mL microwave vial. This mixture was stirred for 30 minutes followed by heating at 150 °C for 20 min at a stirring rate of 600 rpm in a microwave furnace set at a pressure of 20 bars. The bright red product was filtered, washed with 50 mL of water, ethanol, CS_2_ and ether and dried in air at 60 °C.

#### 4.2.2 Synthesis of Na_2_[Mo_3_S_13_]

The as-prepared (NH_4_)_2_[Mo_3_S_13_] was used to synthesize Na_2_[Mo_3_S_13_] following a reported method,^37^ wherein 250 mg of (NH_4_)_2_[Mo_3_S_13_] was dissolved in 40 mL of 1% NaOH solution and stirred for 2 hours under vacuum. This solution was then filtered into 10% NaCl solution and kept for 12 hours to allow for the formation of a Na_2_[Mo_3_S_13_] precipitate. The bright red product was dried in air at 60 °C and stored in a desiccator.

#### 4.2.3 Synthesis and protonation of GCN

This work employed thermally expanded graphitic carbon nitride (GCN) reported and characterized previously.^27,38^ Briefly, a certain quantity of dicyandiamide was loaded into a sealed quartz crucible and placed in a microwave muffle furnace, Phoenix™ (CEM Corporative). The temperature was then gradually raised at 2 °C min^−1^ until it reached 450 °C and kept for 30 min. Subsequently, the temperature was further increased from 450 °C to 550 °C at the same rate (2 °C min^−1^) and held for 60 min under an air atmosphere. The collected sample (labeled as bulk) was washed and dried at 100 °C. To enhance the specific surface area of the photocatalyst, a second thermal treatment at 500 °C was applied for 2 h to the bulk material, resulting in the GCN photocatalyst with nearly a 15-fold increase in the specific surface area (from 7 to 105 m^2^ g^−1^).

The protonation of GCN was carried out following a procedure already reported^34^ wherein GCN was dispersed in 10 mL of 37% HCl solution and stirred for 4 hours at room temperature. The solution was then filtered and washed with water until neutral pH was achieved. The as-modified sample denoted as H-GCN was dried in air at 105 °C overnight.

#### 4.2.4 Synthesis of Mo_3_/GCN composites

The composites of the Na_2_[Mo_3_S_13_] cluster with GCN were synthesized by a method reported previously.^22^ Briefly, GCN was dispersed in methanol and sonicated for 2 h. The Na_2_[Mo_3_S_13_] solution in methanol (10 wt% with respect to GCN mass) was then added to the GCN suspension and kept for stirring overnight. After 24 h the composites were filtered and washed with excess methanol followed by drying in air at 60 °C.

### 4.3 Methods

UV-vis spectroscopy was performed on a Jasco V670 UV-vis spectrometer. The samples were prepared in methanol and aqueous methanol (1 : 1 vol.) solution with a concentration of 0.05 mM; UV-vis spectra were recorded in absorbance mode. Absorption spectra of powdered samples were measured by solid-state *via* diffuse-reflectance spectroscopy (DRS) using MgSO_4_ and GCN as references.

ATR-FTIR spectra of the samples were recorded *via* a PerkinElmer FTIR Spectral UATR-TWO with a Spectrum Two Universal ATR (Single Reflection Diamond) instrument. Powdered samples were directly loaded onto the sample holder and the spectra were recorded in the region of 4000–400 wavenumbers (cm^−1^). Raman measurements were performed with a WITec alpha 300 RSA+ Raman microscope equipped with a 488 nm excitation laser (532 nm) maintaining the laser intensity at 5 mW.

The quantitative elemental analysis of the samples was performed with X-ray photoelectron spectroscopy (XPS) using a custom-built SPECS XPS-spectrometer equipped with a monochromatized Al-K_α_ X-ray source and a hemispherical WAL-150 analyzer (acceptance angle: 60°). To improve the sensitivity of the measurements, Mo_3_/TiO_2_ samples were prepared and investigated in the form of thin-films (see Additional methods in the ESI†). This was followed by wet impregnation of the Na_2_[Mo_3_S_13_] clusters from methanolic solutions. For a single XPS measurement, pass energies of 100 eV and 30 eV and energy resolutions of 1 eV and 100 meV were used for survey and detailed spectra, respectively (excitation energy: 1486.6 eV, beam energy and spot size: 70 W onto 400 μm, angle: 51° to sample surface normal, base pressure: 5 × 10^−10^ mbar, and pressure during measurements: 2 × 10^−9^ mbar). Data analysis was performed using CASA XPS software, employing transmission corrections (as per the instrument vendor's specifications), Shirley backgrounds and Scofield sensitivity factors. Charge correction was applied so the adventitious carbon peak (C–C peak) was shifted to 284.8 eV binding energy (BE). All content values shown are in units of relative atomic percent (at%), where the detection limit in survey measurements usually lies around 0.1–1 at%, depending on the element.

Quantitative determination of the [Mo_3_S_13_]^2−^ cluster loadings was performed by X-ray fluorescence spectroscopy in total reflection geometry (TXRF) using an ATOMIKA 8030C X-ray fluorescence analyzer (Atomika Instruments GmbH, Oberschleissheim, Munich, Germany). The X-ray tube was employed at 50 kV and 47 mA and the selected excitation source was the continuous spectrum of tungsten monochromatized at 35 keV. The samples were excited for 100 s and a Si(Li)-detector was used for X-ray acquisition. The samples were prepared by suspending 1 mg in 1 mL H_2_O for 10 minutes (*c* = 1 mg mL^−1^). 10 μL of a 1000 ppm Yttrium internal standard (for quantification) were added to the suspension, which was subsequently vortexed for 1 min. 5 μL of the suspension with the internal standard were drop cast onto a clean quartz reflector, which was then dried for 5 minutes on a hot plate. After this time, the residue was sealed with 5 μL of a 1% PVA solution and dried for another 5 minutes on a hot plate. The absolute amounts of Mo with respect to Y were then quantified based on the calibration curve and the proportion of Mo (K-line) and Y peak (K-line) areas. This calculation yielded real [Mo_3_S_13_]^2−^ loading values presented in Table 1, considering the undisturbed stoichiometry of Mo to S.

Scanning electron microscopy (SEM) images were acquired using an FEI Quanta 250 FEG scanning electron microscope to obtain visual information on the morphology of the samples. Typically, an acceleration voltage of 10 kV and secondary electron detection mode were used.

High-angle annular dark field (HAADF) STEM imaging and energy dispersive X-ray spectroscopy (EDS) were performed by using a Titan Cubed G2 60-300 (TEM/STEM, FEI Co., now Thermo Fisher Scientific) operated at 300 kV. This microscope has an aberration corrector for STEM (DCOR, CEOS), four-quadrant windowless super-X SDD (silicon drift detector) system. The probe current was ∼60 pA for STEM observation as well as EDS. The convergence semi-angle of the electron probe was 18 mrad. The typical probe diameter was less than 0.1 nm. Forward scattered electrons in an angular range from 38 to 184 mrad were detected using a HAADF detector for STEM imaging.

Powder X-ray diffraction (XRD) of (NH_4_)_2_[Mo_3_S_13_] and Na_2_[Mo_3_S_13_] was performed using an XPERT II: PANalytical XPert Pro MPD (*Θ*–*Θ* diffractometer) for the *ex situ* experiments. The sample was placed on a Si sample holder and irradiated with a Cu X-ray source (8.04 keV, 1.5406 Å). The signals were then acquired with Bragg–Brentano *Θ*/*Θ*-diffractometer geometry ranging from 5° to 80° degrees using a semiconductor X'Celerator (2.1°) detector. XRD analysis of GCN powder was carried out on a PANalytical X'Pert MPD equipped with an X'Celerator detector and a secondary monochromator (Cu Kα *λ* = 0.154 nm, 50 kV, 40 mA; data recorded at a 0.017° step size, 100 s per step).

Steady state photoluminescence (PL) measurements were performed using a PicoQuant FluoTime 300 spectrophotometer. A Xe arc lamp (300 W power) was the excitation source, coupled with a double-grating monochromator. The detection system was composed of a PMA Hybrid 07 detector along with a high-resolution double monochromator. The PL properties of homogeneous solutions were observed using 445 nm excitation wavelength whereas 370 nm light excitation was used to probe the PL spectra of heterogeneous catalysts suspended in water; the concentration of each reaction component was set to mimic those from the corresponding HER experiments. The Mo_3_/GCN samples were prepared by dispersion of the composite in water (0.5 mg mL^−1^), sonication for 30 min, and centrifugation for 30 min. The supernatant solution was diluted with water and 0.1 M ascorbic acid. The data were collected and later fitted using EasyTau2 software. The Stern–Volmer treatment was applied to extract the bimolecular rate constant based on the [Ru(bpy)_3_]^2+^ lifetime of 198.58 ns measured at 615 nm.

### 4.4 Photocatalytic experiments

The visible-light-driven hydrogen evolution experiments were carried out using a 5 mL batch reactor equipped with a monochromatic LED light source (445 ± 13 nm, Thorlabs SOLIS). For the experiments in the homogeneous phase, the reactor was filled with a 2 mL of reaction mixture which comprises 1 : 1 MeOH/H_2_O containing [Ru(bpy)_3_]^2+^ (bpy stands for 2,2′-bipyridine) as a photosensitizer (0.645 mM), l-ascorbic acid (H_2_A) as a proton donor (0.1 M), and the corresponding catalyst Na_2_[Mo_3_S_13_]·H_2_O (50 μM). For the heterogeneous hydrogen evolution experiments the reactor was filled with 2 mL of water containing 0.5 mg mL^−1^ of the Mo_3_/GCN photocatalyst and 0.1 M sacrificial donor (0.1 M ascorbic acid (H_2_A) and triethanolamine (TEOA), details in Fig. S7 and S8d†). Exposure to ambient light was minimized during the solution mixture preparation and transfer to the reactor. The reaction volume was purged with Ar (15 mL min^−1^) for 10 min to ensure the removal of headspace and dissolved oxygen prior to the start of the reaction. The temperature of the reactor was maintained at 15 °C with a water-cooling system. The reaction mixture was stirred at 600 rpm. The H_2_ produced was monitored by sampling the reactor headspace (200 μL) and analyzing its composition *via* gas chromatography (Shimadzu GC 2030) equipped with a barrier ionization discharge detector and a Micropacked-ST column using helium as a carrier gas. Injections were performed with an interval of 30 minutes. Calibration was performed using a range of H_2_ in argon gas mixtures. A set of blank experiments in the absence of [Mo_3_S_13_]^2−^ was conducted otherwise mimicking the conditions of both the homogeneous and heterogeneous HER tests resulting in negligible (<0.1 nmol) amounts of H_2_ generated.

#### 4.4.1 HER stability experiments

Long-term HER experiments for both homogeneous (Mo_3_/Ru) and heterogeneous (Mo_3_/GCN) systems were performed using the purging-degassing method to investigate and compare stability of both. For both the systems, the photocatalytic reaction solutions (for Mo_3_/Ru, 50 μM of Mo_3_, 0.45 mM of [Ru(bpy)_3_]^2+^ in 0.1 M of H_2_A and for Mo_3_/GCN, 10Mo_3_/GCN in 0.1 M H_2_A) were purged with Ar for 10 min. The reaction mixture was then illuminated for 1 h with an LED at 445 nm and sampling was performed from the headspace for H_2_ quantification. The headspace was then degassed and purged again for 10 min before illuminating for 1 more hour. This process was repeated five times to monitor the stability of both the systems.

### 4.5 TON, TOF, and AQY calculation

The H_2_ concentrations in ppm (derived from the chromatograms) were converted to μmol and turnover numbers (TONs – expressed per [Mo_3_S_13_]^2−^ species) based on reactor parameters and the ideal gas equation. Initial turnover frequencies (TOFs) were calculated after 10 minutes of illumination (in most of the cases a close to linear H_2_ evolution trend within the first 60 minutes of the HER was observed). The calculation of the apparent quantum yield (AQY) values considered the ratio between the number of reacted electrons and the number of photons absorbed by the reaction solution.^39,40^ The latter was extracted using a power meter PM100D (Thorlabs) by measuring photon flux at the reactor position (12 mW cm^−2^). To estimate the number of photons absorbed by the reaction solution we first measured intensity of light that reached the detector after passing through the reactor filled with pure solvent and then measured intensity of light that reached the detector after passing through the reactor filled with the Mo_3_/Ru photocatalyst dissolved in the solution. The difference between the values indicated the amount of light that has been trapped by Mo_3_/Ru.

## Author contributions

Conceptualization, SB and AC; methodology, SB and AC; formal analysis, SB, JS, and PA; investigation, SB, JS, PA, HS, MJS and ESS; resources, DE; data curation, SB; writing: original draft preparation, SB; writing: review and editing, SB, JLF and AC; visualization, SB; supervision, CGS, JLF, DE and AC; project administration, AC; funding acquisition, CGS, JLF and AC. All authors have read and agreed to the published version of the manuscript.

## Conflicts of interest

There are no conflicts of interest to declare.

## Supplementary Material

SE-008-D3SE01658G-s001
