# Supplementary material for: A thiomolybdate cluster for visible-light-driven hydrogen evolution: comparison of homogeneous and heterogeneous approaches
Source: Sustain Energy Fuels. 2024 Feb 1;8(6):1225–35. doi: 10.1039/d3se01658g (PMC10929693; doi:10.1039/d3se01658g)
Supplement: SE-008-D3SE01658G-s001 [file SE-008-D3SE01658G-s001.pdf]

Electronic Supplementary Material (ESI) for Sustainable Energy & Fuels.
This journal is © The Royal Society of Chemistry 2024

Electronic Supporting Information (ESI)

Thiomolybdate Cluster for Visible-Light Driven Hydrogen Evolution: Comparison of Homogeneous and Heterogeneous Approaches

Samar Batool[1], Jasmin S. Schubert[1], Pablo Ayala[1], Hikaru Saito[2], Maria J. Sampaio[3,4], Eliana S. Da Silva[3,4,#], Cláudia G. Silva[3,4], Joaquim L. Faria[3], Dominik Eder[1], Alexey Cherevan[1]*

[1] *TU Wien, Institute of Materials Chemistry, Getreidemarkt 9/BC/02, 1060, Vienna, Austria*

[2] *Institute for Materials Chemistry and Engineering, Kyushu University, 6-1 Kasugakoen, Kasuga, Fukuoka 816-8580, Japan*

[3] *LSRE-LCM – Laboratory of Separation and Reaction Engineering – Laboratory of Catalysis and Materials, Faculty of Engineering, University of Porto, Rua Dr. Roberto Frias, 4200-465 Porto, Portugal*

[4] *ALiCE – Associate Laboratory in Chemical Engineering, Faculty of Engineering, University of Porto, Rua Dr. Roberto Frias, 4200-465 Porto, Portugal*

# current address: Departament de Química, Universitat Autònoma de Barcelona, Cerdanyola del Vallès, 08193 Barcelona (Spain)

Contents:

1. Materials characterization 3
2. Choice of photosensitizers and illumination conditions 6
3. Optimization of HER conditions 6
4. Photoluminescence emission spectroscopy 8
5. Reloading experiments 10
6. Postcatalytic characterization 11
7. References 12

1. Materials characterization

XRD and FTIR of the Mo-Clusters

Successful formation of $[Mo_3S_{13}]^{2-}$ clusters was confirmed using FTIR spectroscopy. Figure S1a shows the FTIR spectra of $Na_2[Mo_3S_{13}]$ revealing three peaks at 542 cm^{-1}, 505 (510/501 doublet) cm^{-1}, and 458 cm^{-1} in the fingerprint region that correspond to bridging, terminal, and apical sulfur ligands in the cluster framework.[1,2] Additionally, only OH bending and stretching vibrations are observed at ~1600 cm^{-1} and ~3300 cm^{-1}, which correspond to residual and crystalline water. The purity of the product (e.g. complete cation exchange) is manifested by no extra peaks observed in the IR spectrum. The purity and crystallinity of the $(NH_4)_2[Mo_3S_{13}]$, $Na_2[Mo_3S_{13}]$, and GCN were further confirmed by powder XRD spectra (Figure S1b) that match well with the literature and the database.[1–3]

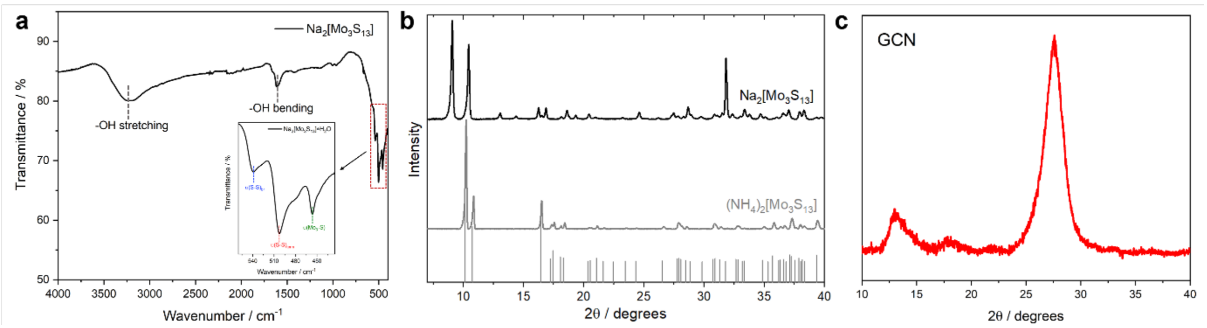

Figure S1 | (a) IR Spectra of $Na_2[Mo_3S_{13}]$, and XRD spectra of **(b)** $Na_2[Mo_3S_{13}]$ and $(NH_4)_2[Mo_3S_{13}]$, **(c)** GCN powder.

DRS

Optoelectronic properties of as-prepared (GCN) and protonated (H-GCN) carbon nitride (details in Experimental section) have been investigated using diffuse-reflectance spectroscopy (DRS). Figure S2a shows as-obtained profiles that highlight strong absorption of both GCN materials in visible-light range (400-700 nm). Tauc analyses (Figure S2b) reveal the band gap of ca. 2.75 eV, which corresponds well to the literature.[4]

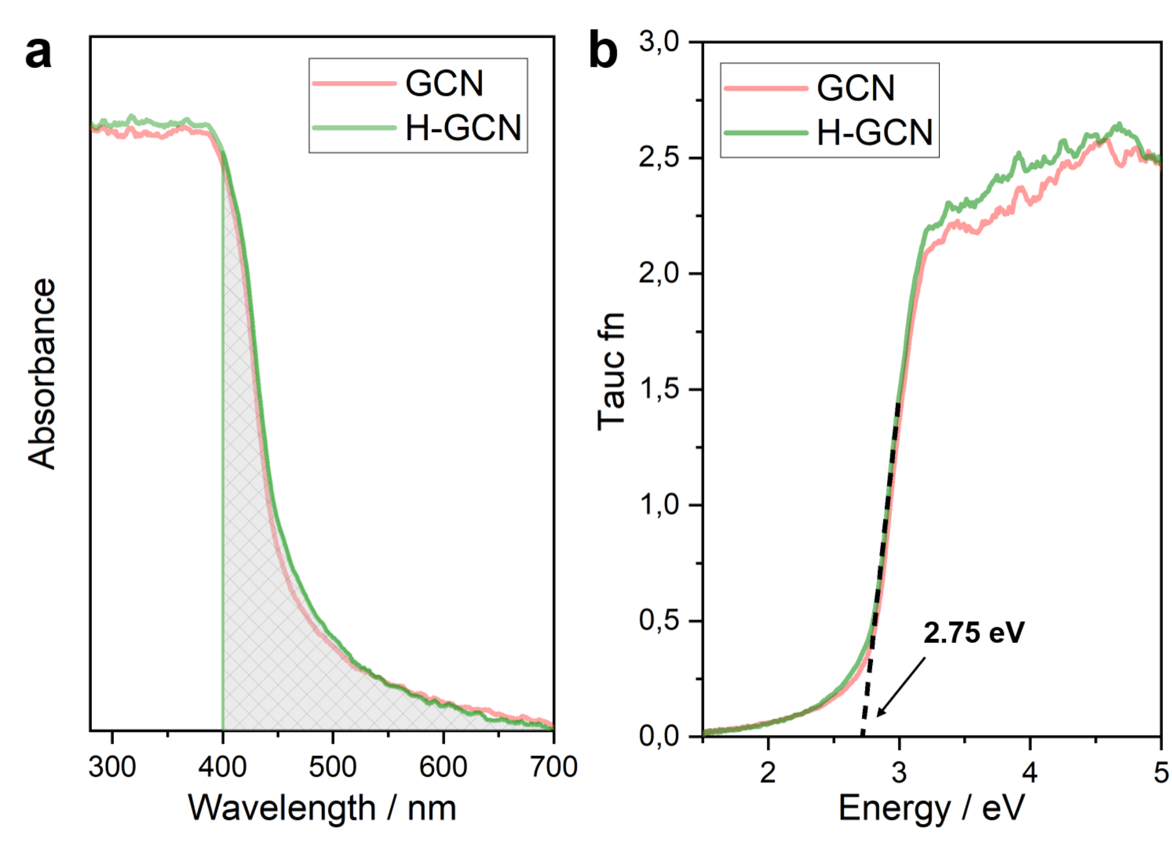

Fig. S2 | DRS plots of **(a)** GCN and H-GCN powders, **(b)** Tauc plots of bare GCN and H-GCN. Shaded area corresponds to the range of visible-light absorption (i.e. >400 nm).

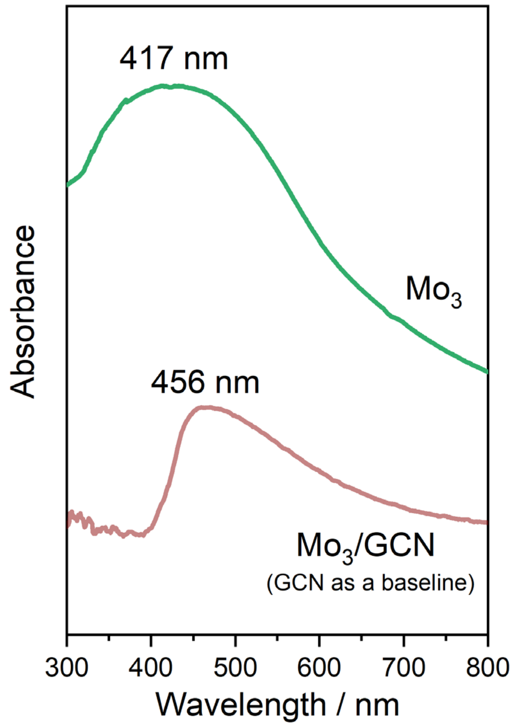

Fig. S3 | DRS plots of $[Mo_3S_{13}]^{2-}$ (Mo_3) and $10Mo_3$/GCN composite, the latter is measured taking GCN as a baseline. The broad peak centered at ~456 nm is the qualitative indication for the presence of Mo_3 on the surface of GCN. The quantitative assessment of the loading values is performed via TXRF (Table 1).

FTIR of Mo_3/GCN

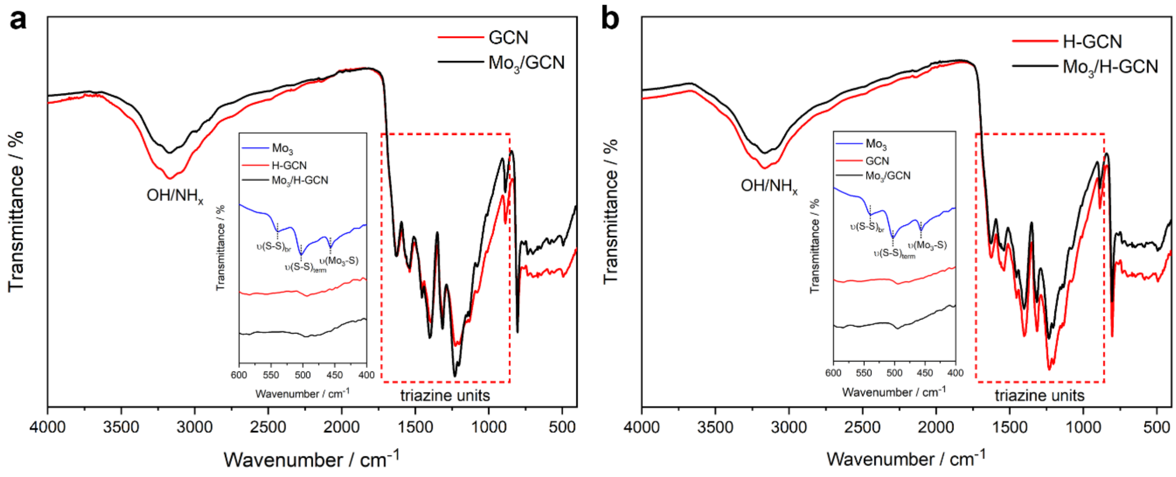

Fig. S4 | FTIR spectra of **(a)** GCN and $10Mo_3$/GCN, **(b)** H-GCN and $10Mo_3$/H-GCN. Insets from 600-400 cm-1 shows the comparison of signature bands of $[Mo_3S_{13}]^{2-}$ (Mo_3) with GCNs and Mo_3/GCNs.

XPS of Mo_3 and Mo_3/GCN

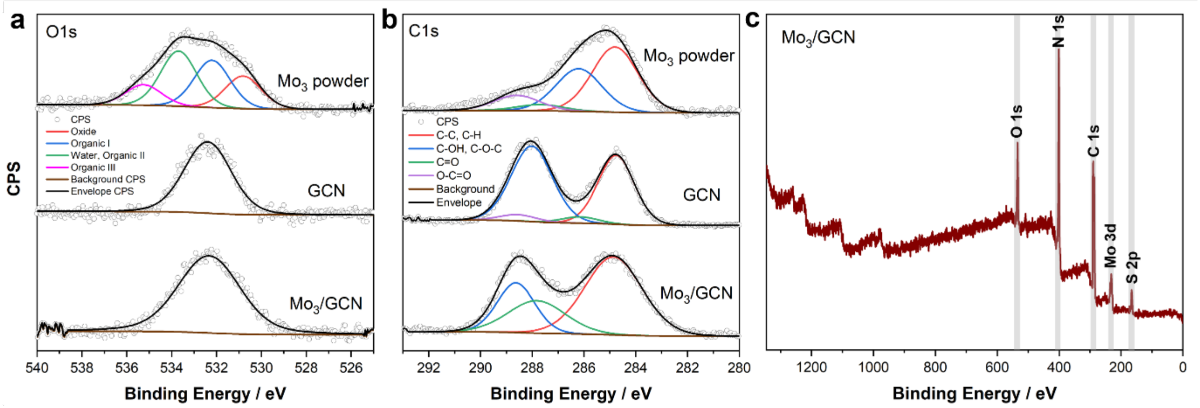

Fig. S5 | XPS spectra **(a)** O1s, **(b)** C1s, of pure $[Mo_3S_{13}]^{2-}$ (Mo_3) clusters, bare GCN, and Mo_3/GCN system. and **(c)** Survey spectra of Mo_3/GCN (see details in experimental section).

2. Choice of photosensitizers and illumination conditions

Comparison of Mo_3/GCN and Mo_3/Ru in terms of excitation source

First, we verified if the heterogenized $[Mo_3S_{13}]^{2-}$ clusters of the Mo_3/GCN composite are able to promote the desired HER under visible-light illumination. Figure 1b compares absorption spectra of the GCN (measured in reflectance mode) and $[Ru(bpy)_3]^{2+}$ (measured in transmission mode) compared to the output spectrum of the visible-light emitter used in this work to evaluate light-driven HER performance of both Mo_3/GCN and Mo_3/Ru. The band gap value of GCN can be estimated to around 2.75 eV (see Figure S2), which allows 445 nm photons to trigger band-to-band excitation (C 2p to N 2p) and generate an electron-hole pair. We note, however, that – compared to the extent of the $[Ru(bpy)_3]^{2+}$ absorption – only edge-to-edge transition in GCN is likely to be triggered by the light source leaving the generated electron-hole pairs with little-to-no overpotential for further reaction.

3. Optimization of HER conditions

Table S1. Optimization of parameters for hydrogen evolution reaction under homogeneous conditions. The concentration of $[Ru(bpy)_3]PF_6$ photosensitizer was kept 0.645 mM for all the experiments mentioned in the table and the reaction mixture was illuminated for 30 min with LED lamp 445 nm.

$[Mo_3S_{13}]^{2-}$ concentration	Solvent	Sacrificial donor	H_2 produced (ppm)	Apparent quantum yield (AQY, %)
10 µM	ACN/H_2O (9:1)	Ascorbic acid (0.1M)	247	0.086
50 µM			515	0.178
10 µM	MeOH/H_2O (9:1)		1100	0.380
50 µM			1508	0.520

*ACN : Acetonitrile, MeOH : Methanol

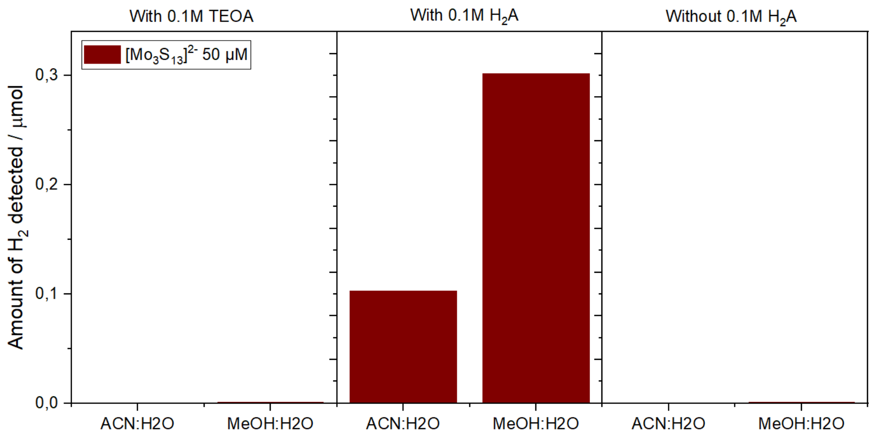

Fig. S6 | Homogeneous HER experiments with and without the addition of electron donor in the photocatalytic system. Conditions: 50 µM $[Mo_3S_{13}]^{2-}$, 0.645 mM $[Ru(bpy)_3]^{2+}$, 0.1M H_2A in MeOH:H_2O (9:1) and ACN:H_2O (9:1) solvent system which is illuminated for 30 min by LED lamp 445 nm.

Overall, several factors are in control of hole scavenging capability of these electron donors (MeOH, TEOA, H_2A): their polarity, which defines the degree of interaction with the photosensitizer (be it a molecule, ion or heterogeneous surface) and their redox properties, which define the thermodynamics of their oxidation.[5–7] However, ascorbic acid (H_2A) produces an intermediate during the hole scavenging process which is reported to oxidize Ru-based PS and therefore reduces the overall hydrogen evolution performance.[5,8] This makes the stability of the Mo_3/Ru photosystem questionable as Ru-PS degrades with increase in illumination time as well as it recombines with oxidized intermediate of ascorbic acid promoting charge recombination. However, when assessing overall HER performance of the photosystem, one should also consider other less direct contributions. First of all, the choice of the solvent system and the associated dielectric constant of the reaction medium affect the final availability of sacrificial donor as well as the efficiency of the charge transfer process.[6] Besides this, the mechanism of electron donor oxidation (via hole trapping) often involves intermediate species that may affect the reaction in a number of ways e.g. act as recombination centers.[5]

4. Photoluminescence emission spectroscopy

Role of sacrificial agent

To verify the role of H_2A as an electron donor in our HER experiments performed with the Mo_3/GCN photosystem, we conducted PL quenching experiments using $[Mo_3S_{13}]^{2-}$-free and $[Mo_3S_{13}]^{2-}$-loaded GCN suspensions in water in and without the presence of H_2A. The reason for this is that the two-electron oxidized species of ascorbate (i.e., dehydroascorbate) as well as radicals generated from H_2A are known to react with the reduced from of the ruthenium dye. Despite the first reaction is slowed by its bimolecular nature, we still consider both being relevant under our reaction conditions.[8,5] As summarized in Figure S7, the addition of 0.1M H_2A to the GCN suspensions ($[Mo_3S_{13}]^{2-}$-free in *a*, with 1 wt.% $[Mo_3S_{13}]^{2-}$ in *b* and with 10 wt.% $[Mo_3S_{13}]^{2-}$ in *c*) reduce the PL intensities in all cases compared to those obtained in pure water. This result confirms the role of H_2A as an efficient hole acceptor, which leads to a better separation of charge carriers photoexcited in GCN under our reaction conditions.

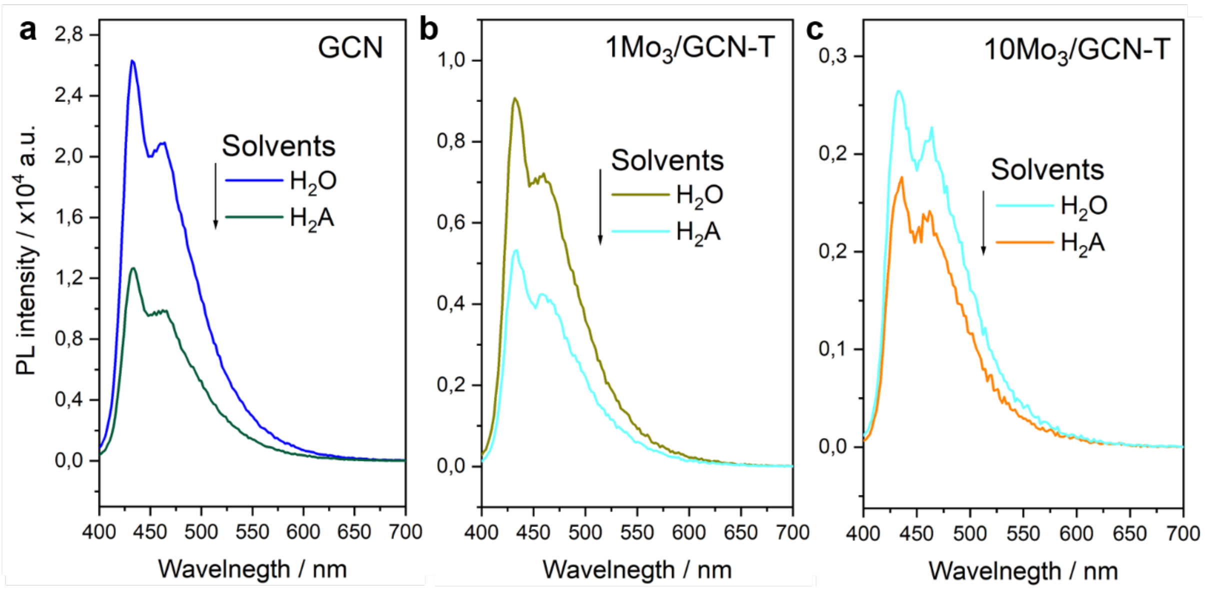

Fig. S7 | PL spectra of **(a)** GCN, **(b)** $1Mo_3$/GCN, **(b)** $10Mo_3$/GCN, mimicking the photocatalytic HER experiment (see details in experimental section).

Choice of H_2A concentration

To further justify the choice of H_2A concentration (0.1 M) used in our HER experiments performed with the Mo_3/GCN photosystem, we conducted additional PL quenching experiments. Figure 8d shows that the addition of low concentrations (0-0.05 M) of H_2A to Mo_3/GCN gradually reduces the PL emission intensity of GCN, which suggests that reductive quenching mechanism is in place and it leads to improved electron/hole separation (i.e. thus affording higher HER performances). It is noteworthy, however, that higher concentrations of H_2A (> 0.05 M) do not lead to further quenching (i.e. saturation is reached). This result indicates that hole extraction by H_2A is not a performance-limiting factor at H_2A concentrations above ~0.1 M and that the use of this H_2A concentration can be justified when conducting HER studies.

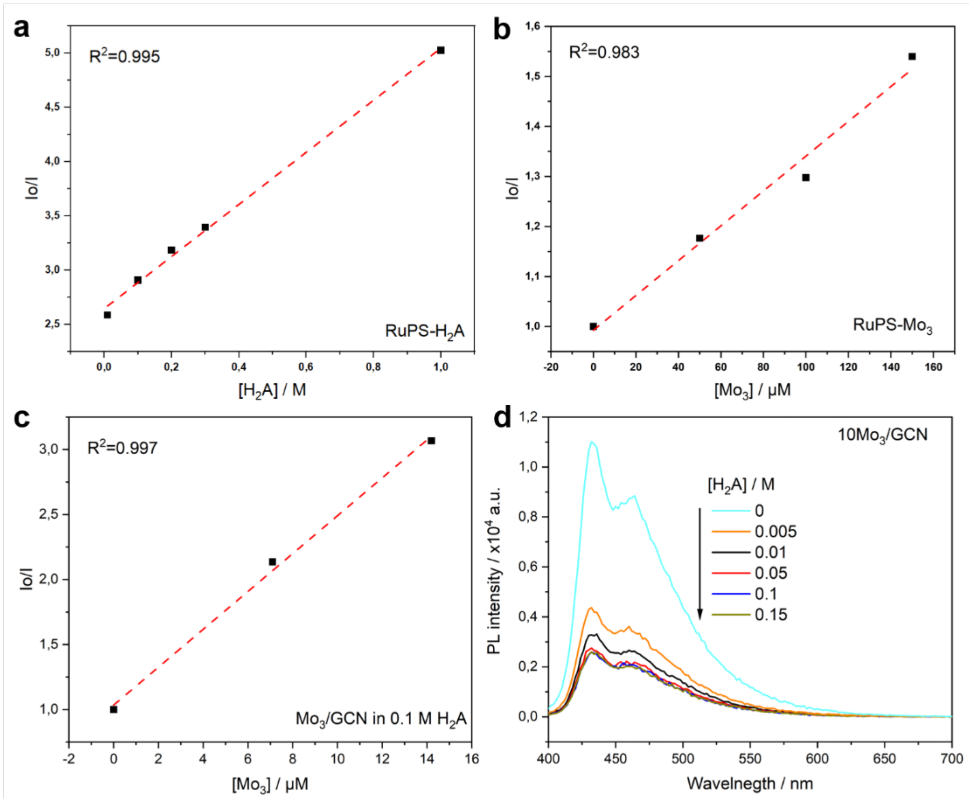

Fig. S8 | Stern-Volmer fitting plots of Mo_3/Ru system with varying concentration of **(a)** H_2A and **(b)** $[Mo_3S_{13}]^{2-}$ **(c)** Mo_3/GCN system with varying concentration of $[Mo_3S_{13}]^{2-}$ (see details in experimental section), **(d)** Mo_3/GCN system with varying H_2A concentration.

5. Reloading experiments

To investigate the stability of $Na_2[Mo_3S_{13}]$ in Mo_3/Ru photosystem, a solution containing 2 mL (4.5:4.5:1) of $[Ru(bpy)_3]^{2+}$ photosensitizer (PS, 0.645 mM in MeOH), the $[Mo_3S_{13}]^{2-}$ catalyst (50 µM in MeOH), and H_2A proton donor (0.1 M in H_2O) solvent mixture was irradiated and the H_2 was detected by gas chromatography until saturated, indicated by a plateau (red curve in Figure S9, the point of 120 min). After this point was reached, the reaction mixture was recharged with 100 µL of a freshly prepared solution of $[Ru(bpy)_3]^{2+}$ photosensitizer (0.645 mM) and 100 µL H_2A to yield 2.2 mL of a reloaded reaction mixture with 545 mM $[Ru(bpy)_3]^{2+}$ PS, 0.9 mM H_2A and 45 µM $[Mo_3S_{13}]^{2-}$, thereby mimicking the initial Mo_3/Ru (1:13) molar ratios of the first HER cycle. Afterwards, sealing, de-gassing and irradiation of the reloaded reaction mixture initiated the second HER cycle (blue curve in Figure S9). In another experiment, after the first HER cycle, the solution was recharged with just the PS (grey curve in Figure S9). Both secondary datasets (grey and blue curves) show that the original activity of the freshly-made Mo_3/Ru photosystem can not be reached when PS (or PS and H_2A) are reloaded. This, in turn, indicates that at least partial degradation of $[Mo_3S_{13}]^{2-}$ takes place along with PS and SA depletion.

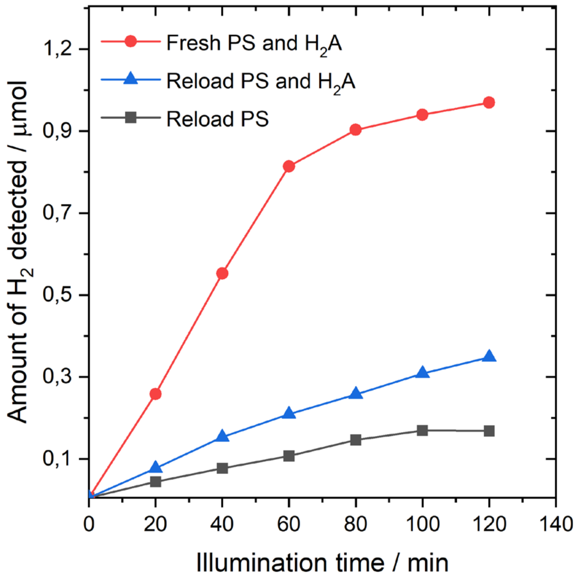

Fig. S9 | Reloading experiments showing the effect of catalytic components on the HER performance. The reaction solution (50 µM $[Mo_3S_{13}]^{2-}$, 0.645 mM $[Ru(bpy)_3]^{2+}$ (PS), and 0.1 M H_2A) after the first HER cycle (red) was recharged with $[Ru(bpy)_3]^{2+}$ (PS) and H_2A (blue) as described above. After purging, the second HER cycle didn't recover the original HER activity. Reloading only the PS after the first HER cycle (grey) accounted for the quarter of original HER activity.

6. Postcatalytic characterization

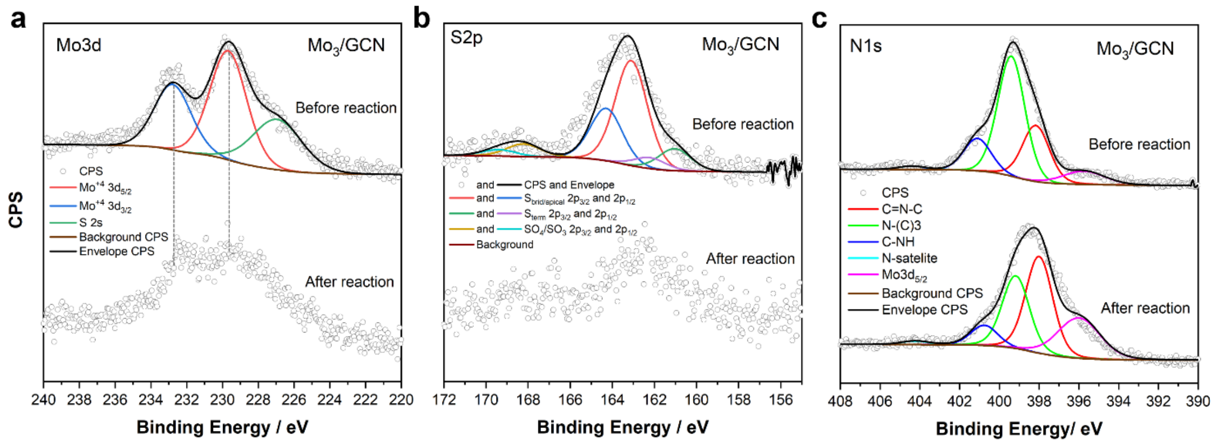

Fig. S10 | XPS spectra **(a)** Mo3d, **(b)** S2p, **(c)** N1s, of the Mo_3/GCN system before and after HER in 0.1M H_2A (see details in experimental section).

Table S2. TXRF of Mo_3/GCN composites before and after hydrogen evolution reaction in ascorbic acid and TEOA sacrificial donors to quantify remaining cluster loadings in wt.%.

Composite	Loading before HER (wt.%)	Loading after HER (wt.%)	
		0.1M H_2A	0.1M TEOA
Mo_3/GCN	3.9	1.7	0.6

7. References

[1] A. Rajagopal, E. Akbarzadeh, C. Li, D. Mitoraj, I. Krivtsov, C. Adler, T. Diemant, J. Biskupek, U. Kaiser, C. Im, M. Heiland, T. Jacob, C. Streb, B. Dietzek, R. Beranek, *Sustain. Energy Fuels* **2020**, *4*, 6085–6095.

[2] S. Batool, S. P. Nandan, S. N. Myakala, A. Rajagopal, J. S. Schubert, P. Ayala, S. Naghdi, H. Saito, J. Bernardi, C. Streb, A. Cherevan, D. Eder, *ACS Catal.* **2022**, *12*, 6641–6650.

[3] A. Müller, E. Diemann, E. Krickemeyer, H. J. Walberg, H. Bögge, A. Armatage, *Eur. J. SOLID STATE Inorg. Chem.* **1993**, *30*.

[4] E. S. Da Silva, N. M. M. Moura, A. Coutinho, G. Dražić, B. M. S. Teixeira, N. A. Sobolev, C. G. Silva, M. G. P. M. S. Neves, M. Prieto, J. L. Faria, *ChemSusChem* **2018**, *11*, 2681–2694.

[5] Y. Pellegrin, F. Odobel, *Comptes Rendus Chim.* **2017**, *20*, 283–295.

[6] M. Wang, S. Shen, L. Li, Z. Tang, J. Yang, *J. Mater. Sci.* **2017**, *52*, 5155–5164.

[7] X. Zhou, Y. Li, Y. Xing, J. Li, X. Jiang, *Dalton Trans.* **2019**, *48*, 15068–15073.

[8] G. Neshvad, M. Z. Hoffman, *J. Phys. Chem.* **1989**, *93*, 2445–2452.
